# Salivary Molecular Spectroscopy with Machine Learning Algorithms for a Diagnostic Triage for Amelogenesis Imperfecta

**DOI:** 10.3390/ijms25179464

**Published:** 2024-08-30

**Authors:** Felipe Morando Avelar, Célia Regina Moreira Lanza, Sttephany Silva Bernardino, Marcelo Augusto Garcia-Junior, Mario Machado Martins, Murillo Guimarães Carneiro, Vasco Ariston Carvalho de Azevedo, Robinson Sabino-Silva

**Affiliations:** 1 Department of Genetics, Ecology, and Evolution, ICB, Federal University of Minas Gerais, Belo Horizonte 312-901, MG, Brazil; felipemorando12345@gmail.com; 2 Department of Clinical Pathology and Dental Surgery, Dental School, Federal University of Minas Gerais, Belo Horizonte 31270-901, MG, Brazil; celiamlanza@gmail.com; 3 Innovation Center in Salivary Diagnostic and Nanobiotechnology, Department of Physiology, Institute of Biomedical Sciences, Federal University of Uberlandia, Uberlandia 38408-100, MG, Brazil; sttephanysb@gmail.com (S.S.B.); marceloagjr@gmail.com (M.A.G.-J.); 4 Laboratory of Nanobiotechnology “Luiz Ricardo Goulart”, Biotechnology Institute, Federal University of Uberlandia, Uberlandia 38408-100, MG, Brazil; mariomm1988@yahoo.com.br; 5 Faculty of Computing, Federal University of Uberlandia, Uberlandia 38408-100, MG, Brazil; mgcarneiro@ufu.br

**Keywords:** saliva, biomarker, amelogenesis imperfecta, FTIR, spectroscopy, artificial intelligence, salivary diagnostics

## Abstract

Amelogenesis imperfecta (AI) is a genetic disease characterized by poor formation of tooth enamel. AI occurs due to mutations, especially in AMEL, ENAM, KLK4, MMP20, and FAM83H, associated with changes in matrix proteins, matrix proteases, cell-matrix adhesion proteins, and transport proteins of enamel. Due to the wide variety of phenotypes, the diagnosis of AI is complex, requiring a genetic test to characterize it better. Thus, there is a demand for developing low-cost, noninvasive, and accurate platforms for AI diagnostics. This case-control pilot study aimed to test salivary vibrational modes obtained in attenuated total reflection fourier-transformed infrared (ATR-FTIR) together with machine learning algorithms: linear discriminant analysis (LDA), random forest, and support vector machine (SVM) could be used to discriminate AI from control subjects due to changes in salivary components. The best-performing SVM algorithm discriminates AI better than matched-control subjects with a sensitivity of 100%, specificity of 79%, and accuracy of 88%. The five main vibrational modes with higher feature importance in the Shapley Additive Explanations (SHAP) were 1010 cm^−1^, 1013 cm^−1^, 1002 cm^−1^, 1004 cm^−1^, and 1011 cm^−1^ in these best-performing SVM algorithms, suggesting these vibrational modes as a pre-validated salivary infrared spectral area as a potential biomarker for AI screening. In summary, ATR-FTIR spectroscopy and machine learning algorithms can be used on saliva samples to discriminate AI and are further explored as a screening tool.

## 1. Introduction

Amelogenesis imperfecta (AI) is a genetic condition characterized by impaired amelogenesis and enamel deposition on teeth, and it has as a complex treatment. This rare condition occurs due to mutations in several genes, such as, AMEL, ENAM, KLK4, MMP20, and FAM83H, related to matrix proteins, matrix proteases, cell-matrix adhesion proteins, and transport proteins of enamel [1,2]. The AI clinical phenotype can be classified by a reduced amount of enamel (hypoplasia), deficient calcification (hypocalcification), poor enamel maturation (hypomaturation), or poor mineralization (hypomineralization) [1]. Further, the inheritance of AI can be autosomal recessive (AR), autosomal dominant (AD), X-linked (XL), and X-linked dominant (XLD), which generate an overlapping of clinical symptoms and issues to an adequate diagnosis and management of AI [3]. The prevalence of AI is highly divergent worldwide, and some analyses indicate 1 case per 718 people in Sweden and the other 1 per 14,000 people in the United States [4]. Moreover, the prevalence of AI in several countries, such as Brazil, is unknown.

Other non-exocrine sources mixed in saliva are desquamated epithelial cells, intact and blood cell-derived components, and gingival crevicular fluid [5]. Saliva is a complex biological fluid with over 3000 proteins, thousands of different mRNAs, hundreds of microRNAs, several metabolites, lipids, carbohydrates, and microorganisms. Bearing that saliva collection is a self-collecting, convenient, and noninvasive method [6,7,8], it is a promising alternative for screening or diagnosing genetic diseases. Saliva is derived mainly from acinar cells in salivary glands in a process modified in ductal cells [5,9]. In this context, it was shown that genetic variations in AI genes could be related to changes in calcium and phosphorus salivary levels [10]. The gene ESRRB can be expressed in salivary gland tissue and during enamel development [11], indicating the relationship of classical genes related to enamel with changes in salivary composition. Some salivary proteins were detected only in patients with molar-incisor hypomineralization [12].

A promising alternative to analyzing salivary components by a reagent-free, sustainable, and rapid analysis is the application of the attenuated total reflection fourier transform infrared (ATR-FTIR) [7,13]. This green technology platform can detect vibrational modes derived from salivary components with high sensitivity and specificity [14,15]. ATR-FTIR spectroscopy can provide a pan-omic profile that captures various functional groups from proteins, lipids, nucleic acids, and carbohydrates across multiple omics, including proteomics, lipidomics, and metabolomics [15]. This omics technology can simultaneously offer a molecular composition of numerous components in a single analysis [15]. The salivary spectra in the ATR-FTIR platform can detect lipids; amides I, II, and III of proteins; methyl vibrations from peptides; nucleic acids as RNA; and derivates from carbohydrates and glycans [7,16,17].

Our study aimed to test the hypothesis that some salivary vibrational modes obtained in attenuated total reflection-fourier transform Infrared (ATR-FTIR) coupled with learning-machine algorithms could discriminate AI from control subjects due to changes in salivary components. The present case-control pilot study aimed to compare salivary vibrational modes between AI patients and matched control subjects using ATR-FTIR spectroscopy coupled to linear discriminant analysis (LDA), Random Forest, and supporting vector machine (SVM) algorithms.

## 2. Results

The mean age was similar (*p* > 0.05) in control and AI subjects (18 ± 6 and 16 ± 5 years old, respectively). The gender percentage was similar (*p* > 0.05) for both control and AI subjects. In this context, the gender percentage was 45.4% for males and 54.6% for females in control subjects, and this parameter was 50% for males and 50% for females in AI subjects. In the AI cases, 83.3% presented autosomal dominant as an inherited pattern, and 16.7% did not determine it.

### Blood Plasma Infrared Spectroscopy

**Figure 1:**
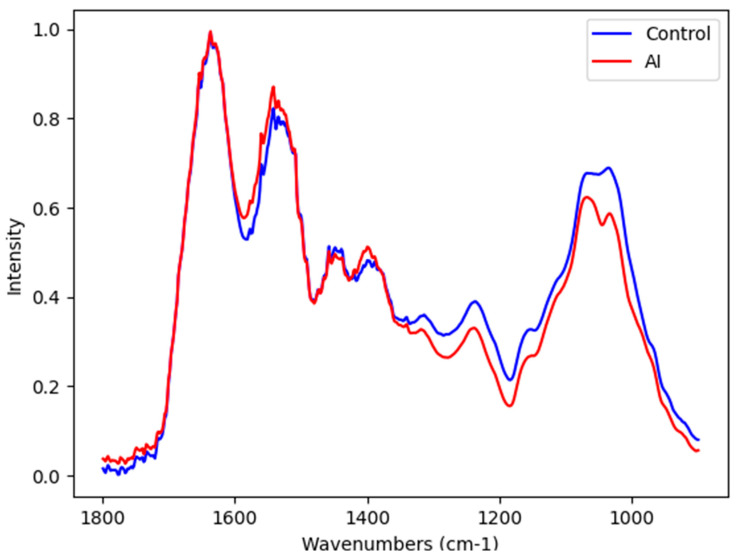
Representative average ATR-FTIR raw spectra (1800–800 cm^−1^) in control (**blue**) and AI (**red**) samples.

The mean infrared spectra normalized by amide 1 in the salivary spectra of control and AI samples were presented in Figure 1. The fingerprint region (1800–900 cm^−1^) can detect salivary components such as proteins, lipids, DNA/RNA, and carbohydrates (Figure 1).

**Figure 2:**
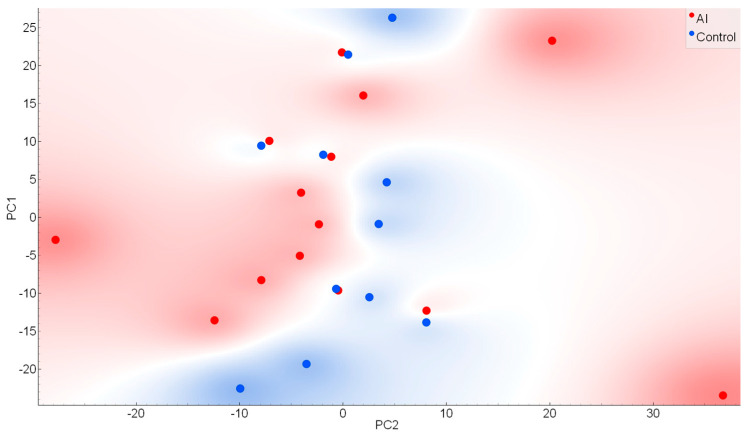
Principal component analysis score plot for two main PCs. Control samples were represented in blue and AI samples in red.

The Principal Component Analysis (PCA) was applied to evaluate the capacity to reduce the dimensional space based on the comparison between the salivary infrared spectra in AI and control subjects. These salivary spectra displayed some areas with visual changes between both classes. The two main principal components (PCs) named PC1 and PC2 explained 49.7% of the cumulative variance (PC1: 30.5% and PC2: 19.2%); these two main PCs are represented in the scores plot (Figure 2). The PC3 represents 18.3%, indicating a total explanation of the cumulative variance of 68% with 3 main PCs in this data exploration phase.

The classification with linear discriminant analysis (LDA), Random Forest, and support vector machine (SVM) algorithms showed the discrimination of salivary infrared spectra applied in AI and control samples. The best discrimination of the LDA algorithm was obtained using pre-processing with rubberband plus normalization by amide I, reaching 82% of sensitivity, 64% of specificity, and 72% accuracy. The classification of salivary infrared spectra by Random Forest with Savitzky-Golay pre-processing also showed an accuracy of 72%, sensitivity of 64%, and specificity of 79% between AI and control subjects. The best discrimination was obtained with the SVM algorithm reaching a sensitivity of 100%, specificity of 79%, and accuracy of 88% between AI and matched-control subjects (Table 1).

**Figure 3:**
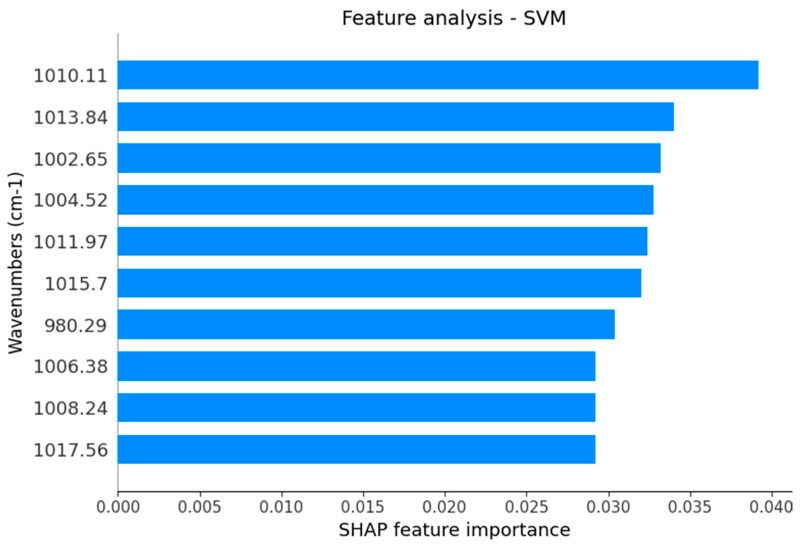
Main wavenumber with respective SHAP feature importance for the best-performing SVM algorithm to discriminate AI and control samples of saliva.

The Shapley Additive Explanations (SHAP) analysis of the best-performing SVM algorithm indicates the main wavenumbers responsible to discriminate spectra from AI and control samples are represented with their respective SHAP feature importance (Figure 2). SHAP is an approach used to explain the quantification of the significance of each feature (wavenumber) regarding a specific model prediction. Here, SHAP measures the impact of the presence or absence of each wavenumber to improve or worsen the accuracy. The feature analysis of the best-performing SVM algorithm indicates the discrimination capability wavenumbers responsible for the best algorithm between AI and control samples is represented in Figure 3. As an outcome, the main wavenumbers with higher SHAP feature importance were 1010 cm^−1^, 1013 cm^−1^, 1002 cm^−1^, 1004 cm^−1^, 1011 cm^−1^, 1015 cm^−1^, 980 cm^−1^, 1006 cm^−1^, 1008 cm^−1^, and 1017 cm^−1^ as the main responsible for distinguishing AI from age- and gender-matched healthy subjects. The tentative molecular assignments of each vibrational mode selected to discriminate AI from age- and gender-matched healthy subjects were described in Table 2 [18,19,20].

## 3. Discussion

The detection of infrared spectral changes in the saliva of AI patients could offer a novel, non-invasive, sustainable, and rapid alternative for assisting dentists in diagnosing AI using saliva samples. This investigation represents the first attempt to evaluate whether ATR-FTIR spectroscopy can effectively differentiate between AI and control saliva samples. Our findings suggest that this portable biophotonic device successfully identified differences in saliva spectra associated with AI, indicating that ATR-FTIR spectroscopy has the potential to serve as a diagnostic platform for future studies on infrared biomarkers in large and multicentric cohorts with control and AI subjects. We envisage that this platform has considerable potential to be used in decentralized point-of-care settings [21], including dental offices with reduced infrastructure.

Although saliva is a complex biofluid with thousands of molecules, the diversity of classes and its unique relative amounts of salivary components can partially discriminate by visual comparison of infrared spectral patterns of the AI. The visual observations of the representative salivary infrared raw spectra from AI and matched-control subjects suggest slight variance in the salivary spectral region 1800–1350 cm^−1^, thus suggesting a similar presence of amide I, amide II, and amide III in salivary proteins [16,19]. Visually, there were clear ATR-FTIR spectral differences in the salivary spectral region 1800–1350 cm^−1^, thus suggesting changes in nucleic acids, proteins/glycoproteins, some minerals, and carbohydrates [16,19].

Hence, a careful exploratory analysis using PCA indicates an intermediate separation between AI and control samples, with a total explained cumulative variance reaching 68% with 3 main PCs. Although the PCA analysis was not the main aim, the partial discrimination with an overlapped distribution of both classes suggests that the discrimination is not simple and needs several wavenumbers to distinguish both classes [19,22,23]. In general, it reinforces the need for machine learning algorithms to improve the accuracy of the test.

Although the diagnosis is frequently performed with clinical examination, novel diagnostic tool alternatives can be effectively applied in the dental office of the public and private healthcare system to prevent several pitfalls in the AI diagnosis. The main pitfalls include the masking of changes in enamel by saliva and dental plaque with inappropriate lighting in the dental office, the presence of caries, attrition, and changes in tooth structure by traumas. Moreover, the high cost of single nucleotide polymorphisms (SNP) significantly reduces confirmation by genetic tests [24,25]. In this context, the present cohort study shows the same accuracy of 72% for LDA and Random Forest using salivary infrared spectra. Furthermore, the SVM algorithm discriminates AI more than matched-control subjects with a sensitivity of 100%, specificity of 79%, and accuracy of 88%. In summary, ATR-FTIR spectroscopy coupled with machine learning algorithms can be viewed as an emerging green technology used in saliva samples to discriminate AI and further explored as an additional screening tool for AI in dental settings.

Subsequently, we examined the main salivary vibrational modes with higher SHAP feature importance for the best-performing SVM algorithm that distinguishes AI from age- and gender-matched healthy subjects. These vibrational modes could potentially be used in a panel of AI infrared spectral markers. One spectral region between 1015–1011 cm^−1^ including three functional groups 1015 cm^−1^, 1013 cm^−1^, and 1011 cm^−1^ related antisymmetric stretching mode of PO4 tetrahedra presumably related to shifts in the chemical structure of hydroxyapatite [20] was indicated by the SHAP analysis to be used by the best-performing SVM algorithm. It can be related to the unbound Ca–O bonds from PO4 functional groups in the enamel which is in contact with saliva [20]. Another spectral region between 1006–1004 cm^−1^, including these two vibrational modes, can be related to changes in sugar moieties from salivary glycoproteins. The SHAP analysis also found that AI can be associated with changes in DNA (vibrational mode at 1017 cm^−1^), stretching C-O deoxyribose in carbohydrates (vibrational mode at 1010 cm^−1^ and 1002 cm^−1^), C = C torsion in salivary proteins (vibrational mode at 1008 cm^−1^), and functional groups of OCH3 in polysaccharides (vibrational mode at 980 cm^−1^) [19]. The change in vibrational mode at 1008 cm^−1^ could be related to changes in salivary proteins as described in AI subjects [12]. Interestingly, the expression of the ESRRB gene in salivary gland tissue and during enamel development [11] suggests a connection between classical genes related to the enamel and a non-canonical expression of these genes in salivary glands.

The claim to apply environmentally friendly technology in non-invasive biofluids for rapid identification of diseases involves multiple factors, and it should be considered in parallel with the presence of some limitations. Infrared spectra use less file size with reduced storage costs compared to the processing data in images while still providing sufficient data for effective faster discrimination with a reduction in training costs. However, one limitation of ATR-FTIR data acquisition is its less intuitive scope to interpret test information [26]. The assistance of machine learning algorithms was addressed here to provide an easy-to-use diagnostic method with fast delivery of results on the decision-making process. Due to the pioneering nature of this study using salivary detection of AI using biophotonic devices, further studies are required to validate the proposed infrared spectral biomarkers to determine the suitability of this green technology for a diagnostic triage of AI. The present study presents limitations by the cohort size, and it comes especially in the analysis of multi-wavenumber predictive models. However, we admit that these present original data could provide significant advances to further larger network analysis focused on exploring these predictive models.

## 4. Materials and Methods

### 4.1. Study Design

This case-control study was conducted in a public dental clinic within the School of Dentistry at UFMG. The cohort studied represents a convenience sample of matched-control samples and AI patients in this referred dental clinic. Inclusion criteria encompassed individuals aged 10 to 30 years old, all with a confirmed diagnosis of AI, who were eligible to participate in the study. The exclusion criteria included the presence of active oral diseases (including active cáries, periodontitis, and xerostomia) and systemic diseases (including hypertension, diabetes mellitus, and chronic kidney diseases). Fourteen (14) patients clinically and radiographically diagnosed with AI and eleven (11) age- and gender-matched healthy controls with similar oral health conditions were recruited from the Department of Dentistry at the Federal University of Minas Gerais. The dental phenotypes of AI were assessed through clinical and radiographic evaluations associated with family histories. Parents, guardians, or subjects gave written informed consent for the enrolment in the present study (CAAE: 59154622.8.0000.5149) following the Declaration of Helsinki guidelines.

### 4.2. Saliva Collection

Saliva samples were collected using slight suction through a soft plastic catheter. No intentional stimulation was used, although the presence of the soft plastic catheter is capable of slightly stimulating the salivary flow. Saliva was collected for two minutes to minimize the stress. The subjects remained comfortably seated in a well-ventilated room during the saliva collection period. After the saliva collection, samples were immediately stored at −80 °C in polypropylene graduated microtubes until the analysis [27,28,29,30]. Participants were requested to abstain from feeding 1 h before the saliva collection. Urine samples were collected from the neonates on the second day of life using a sterile urine collector. The urine samples were transferred to 1.5 mL microtubes and immediately centrifuged (3800 rpm, 5 min, room temperature). The supernatant was collected and stored at −80 °C until analysis [27,29].

### 4.3. Chemical Profile of Unstimulated Saliva by ATR-FTIR Spectroscopy

The infrared salivary spectra were collected using an FTIR Benchtop System Cary 630 FTIR Spectrometer (Agilent Technologies, Santa Clara, CA, EUA) combined with a micro-attenuated total reflectance (ATR) device between 4000 cm^−1^ and 650 cm^−1^. The ATR accessory employs a type IIa diamond crystal as the interface between the sample and the infrared [21,30]. Saliva samples were inserted into an aluminum disc for high-throughput analysis to perform infrared analysis. The high-throughput analysis is critical to ATR-FTIR implementation in biomedical laboratory settings for clinical analysis. The slight background promoted by the infrared spectral interference of aluminum and its sustainable characteristics offers a potential low-cost device for high-throughput analysis. Ten μL of saliva were dried on a hot plate at 80 °C under aluminum devices for 5 min. All IA and control samples were recorded in duplicate, and the mean was used in the analysis. The air spectrum was considered as a background in all ATR-FTIR analyses for atmospheric correction. Salivary pellicle spectra and background were captured with 4 cm^−1^ resolution and 32 scans [7,13,14].

### 4.4. Chemometric Analysis

To perform the principal component analysis (PCA), infrared spectra were processed in the software Orange 3.35.0 based on a Python 3 programming language. PCA is a versatile statistical method for reducing the number of variables in a multidimensional data set. PCA is classified as an unsupervised method capable of indicating the discrimination of different data sets [31]. The variables (*n*) are reduced in a few components (PCs) that maximally explain the variance of all initial variables in the form of scores. The primary graphical result is present in a biplot pattern using the major components to differentiate samples from distinct classes [32,33].

### 4.5. Spectra Data Evaluation Procedures

The data analysis of salivary infrared spectra was divided into pre-processing and classification. The pre-processing stage consisted of aggregation, attribute selection, and data transformation. The arithmetic mean of the three spectral readings of each sample was performed in aggregation. The spectral data were truncated to select only the fingerprint region (1800–900 cm^−1^). Furthermore, we applied selected preprocessing parameters before applying machine learning algorithms [21].

The classifications with each pre-processing parameter were tested with machine learning algorithms associated with linear discriminant analysis (LDA), random forest, or support vector machine algorithms (SVM). For the validation process, we considered a leave-one-out procedure, in which one sample per time is considered exclusively as test data and others as training data. Such a procedure is repeated n times, in which n is the number of samples in the dataset, so it can provide a frank account of the predictive performance of each classification configuration. The predictive performance of the LDA, Random Forest, and SVM were obtained after the leave-one-out procedure [21]. The sensitivity, or true positive rate, is the proportion of positives (AI samples) correctly classified, and the specificity, or true negative rate, is the proportion of negatives (controls) correctly classified. Accuracy is the total number of samples correctly classified considering true and false negatives [21].

These parameters are calculated as follows:

Accuracy (%) = [(TP + TN)/(TP + FP + TN + FN)] × 100

Sensitivity (%) = [TP/(TP + FN)] × 100

Specificity (%) = [TN/(TN + FP)] × 100

where TP stands for true positives, TN for true negatives, FP for false positives, and FN for false negatives, and accuracy (defined as the total number of samples correctly classified) [21,26].

### 4.6. Statistical Analysis for Epidemiological Characteristics

The age and gender proportions were analyzed using the GraphPad Prism 8 software. The Shapiro-Wilk test was used to assess the normality of the data. The data of age were normally distributed, then it was analyzed by the t-test and reported as the mean (±standard deviation). Gender data were analyzed by the Chi-square test and reported as the number of cases (percentage). *p* values less than 0.05 were considered with statistical significance.

## 5. Conclusions

The present study provides the first indication that the molecular changes of saliva from AI and control-matched subjects present potential to be detected using a reagent-free and sustainable system based on ATR-FTIR spectroscopy. As an additional novelty of this approach, we showed that AI could be used as a screening platform using minimal saliva samples with fast delivery of results based on SVM algorithms.

## Figures and Tables

**Table 1:** Machine learning algorithms are applied to classify control and AI samples in salivary spectra.

Algorithm (Spectral Area)	Pre-Processing	Sensitivity	Specificity	Accuracy
**Linear Discriminant Analysis (LDA)** 1800–900 cm^−1^	rb + amide I *	82%	64%	72%
	Savitzky–Golay	64%	57%	60%
**Random forest** 1800–900 cm^−1^	rb + amide I *	54%	50%	52%
	Savitzky–Golay	64%	79%	72%
**Supporting Vector Machine (SVM)** 1800–900 cm^−1^	rb + amide I *	**100%**	**79%**	**88%**
	Savitzky–Golay	73%	71%	72%

* rb + amide I: pre-processing with rubberband plus normalization by amide I.

**Table 2:** Selected vibrational modes by SVM to discriminate AI from age- and gender-matched healthy subjects and its tentative molecular assignments.

Selected Vibrational Mode	Tentative Assignment	Type of Potential Source
1017 cm^−1^	DNA	DNA
1015 cm^−1^	Antisymmetric stretching mode of PO_4_ tetrahedra	Hydroxyapatite
1013 cm^−1^	Antisymmetric stretching mode of PO_4_ tetrahedra	Hydroxyapatite
1011 cm^−1^	Antisymmetric stretching mode of PO_4_ tetrahedra	Hydroxyapatite
1010 cm^−1^	Stretching C-O deoxyribose	Carbohydrates
1008 cm^−1^	C = C torsion	Proteins
1006 cm^−1^	Sugar moieties from glycoproteins	Glycosylated proteins
1004 cm^−1^	Sugar moieties from glycoproteins	Glycosylated proteins
1002 cm^−1^	Stretching C-O deoxyribose	Carbohydrates
980 cm^−1^	OCH3 (polysaccharides)	Carbohydrates

## Data Availability

The raw data supporting the conclusions of this article will be made available by the authors upon request.
